# Fungal Infection in the Brain: What We Learned from Intravital Imaging

**DOI:** 10.3389/fimmu.2016.00292

**Published:** 2016-08-02

**Authors:** Meiqing Shi, Christopher H. Mody

**Affiliations:** ^1^Division of Immunology, Virginia-Maryland Regional College of Veterinary Medicine, University of Maryland, College Park, MD, USA; ^2^Department of Microbiology, Immunology and Infectious Diseases, University of Calgary, Calgary, AB, Canada; ^3^Department of Internal Medicine, University of Calgary, Calgary, AB, Canada

**Keywords:** intravital imaging, fungi, brain migration, immune cells, meningoencephalitis, *C. neoformans*

## Abstract

Approximately 1.2 billion people suffer from fungal diseases worldwide. Arguably, the most serious manifestation occurs when pathogenic fungi infect the brain, often causing fatal meningoencephalitis. For most fungi, infection occurs *via* the vascular route. The organism must first be arrested in the brain microvasculature and transmigrate into the brain parenchyma across the blood–brain barrier. As a result, host immune cells are recruited into the brain to contain the fungi. However, it remains poorly understood how fungi traffic to, and migrate into the brain and how immune cells interact with invading fungi in the brain. A new era of intravital fluorescence microscopy has begun to provide insights. We are able to employ this powerful approach to study dynamic interactions of disseminating fungi with brain endothelial cells as well as resident and recruited immune cells during the brain infection. In this review, with a focus on *Cryptococcus neoformans*, we will provide an overview of the application of intravital imaging in fungal infections in the brain, discuss recent findings and speculate on possible future research directions.

## Introduction

**Figure 1:**
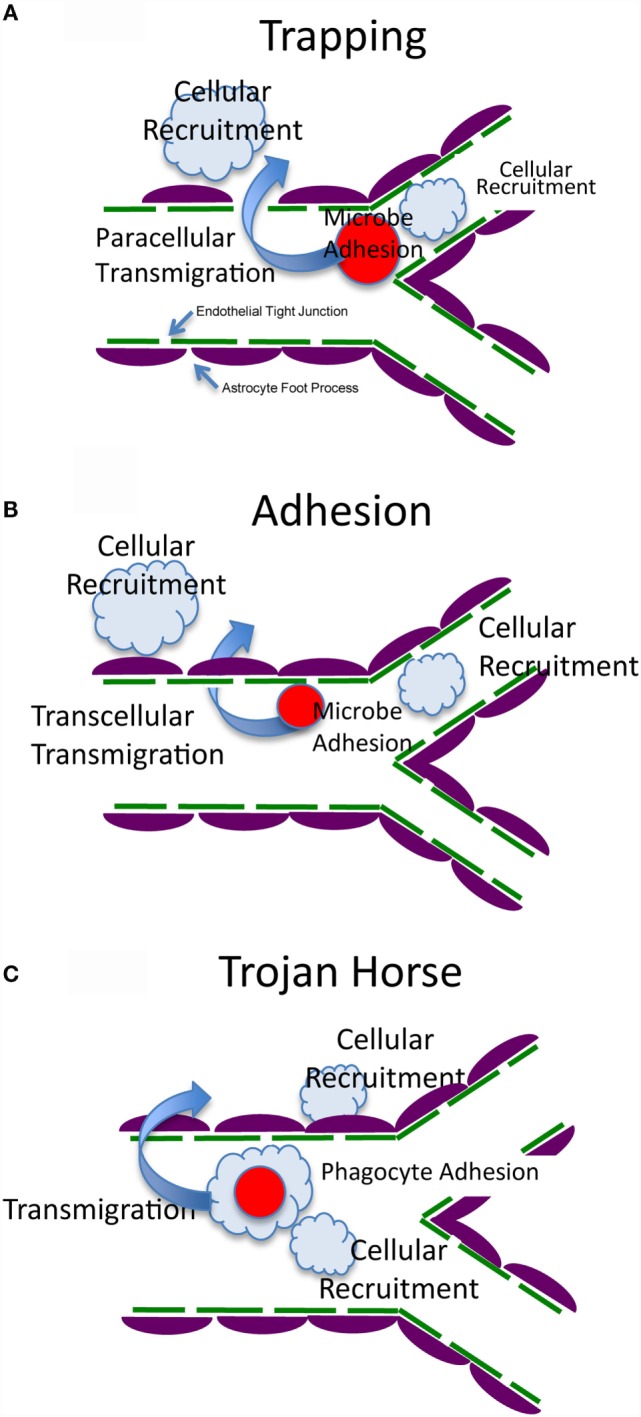
**Possible mechanisms of arrest, transmigration, and resultant host response**. The BBB is formed by brain endothelial cells, which are connected by tight junctions, and astrocyte foot processes that surround the endothelial cells and maintain the integrity of the BBB (2, 3). **(A)** Fungal cells are trapped by vascular constriction with possible sensing and signaling of both cell types (4, 5). This is followed by transmigration that could be by a trans- or paracellular mechanism (paracellular is shown in this panel). Immune and inflammatory cells are recruited to the vascular or extracellular compartment to generate host defense and inflammation. **(B)** Fungal cells adhere directly to the endothelium with possible sensing and signaling of both cell types (6–10). This is followed by transmigration that could be by a trans- or paracellular mechanism (transcellular is shown in this panel). Immune and inflammatory cells are recruited to the vascular or extracellular compartment to generate host defense and inflammation. **(C)** Fungal cells are internalized within a host cell (Trojan Horse) that makes contact with the endothelium, arrests, and generates sensing and signaling of all three cell types (11, 12). This is followed by transmigration that could be by a trans- or paracellular mechanism. Immune and inflammatory cells are recruited to the vascular or extracellular compartment to generate host defense and inflammation.

Infectious meningitis and encephalitis are a major threat to human health, causing high mortality and morbidity throughout the world (1). Following infections, microbes including viruses, bacteria, fungi, and parasites can disseminate from sites of initial infection to the bloodstream. The circulating pathogens become arrested in the brain vasculature, followed by transmigration into the brain parenchyma across the blood–brain barrier (BBB). The BBB is a structural and functional barrier, which maintains the neural microenvironment by regulating the passage of molecules and cells into the brain (2). To date, three mechanisms have been proposed for pathogens to cross the BBB: transcellular migration, paracellular migration, and the Trojan horse mechanism (1). Once pathogens have translocated to the brain parenchyma, they proliferate and cause brain inflammation, often with devastating consequences. There are three fundamental questions in the field (Figure 1): (1) How are pathogens arrested in the brain vasculature? (2) How do pathogens migrate into the brain across the BBB? and (3) How do immune cells respond to the brain infection and do they clear the pathogen or cause inflammation in a constrained intracranial compartment that is highly susceptible to cellular dysfunction and increased pressure?

Modern advances in technology have provided opportunities to better understand host–pathogen interactions. Among them, imaging of organs in living animals, using high-resolution intravital microscopy (IVM), represents a major advance in the field. Using this technique, interactions of pathogens with brain endothelial cells, and their transmigration across the BBB can be directly assessed under flow conditions in real time. In addition, the dynamic interactions of leukocytes with pathogens and their behavior in the brain vasculature and parenchyma can be evaluated in living animals. This is of particular importance, because the extravascular migration of pathogens and their interactions with immune cells are transient and highly dynamic, and investigation of these processes by direct observation using IVM provides insights that cannot be obtained using other techniques.

Of the approximately 300 fungal species that have been reported to be pathogenic to humans (13), *Cryptococcus neoformans, Candida albicans, Histoplasma capsulatum, Coccidioides immitis, Paracoccidioides brasiliensis, Aspergillus* spp., and zygomycetes are among the most common causes of brain or meningeal infections (14–21). In particular, cryptococcal meningoencephalitis is one of the most common infections of the central nervous system and a leading course of HIV-associated mortality globally (16, 18, 22). In recent years, much progress has been made to understand migration of pathogens and immune responses induced by the invading pathogens in the brain using IVM. This review will discuss recent studies that used IVM to address brain infections by a very limited subset of pathogenic fungi (Table 1).

**Table 1:** **Application of intravital imaging to brain infection by fungi**.

Fungi	Animals	Nature of the work	Reference
*C. neoformans*	Mouse	The fungal cell is mechanically trapped in the brain capillary and transmigrates to the brain parenchyma with contributions from urease	Shi et al. (5)
*C. neoformans*	Mouse	Neutrophils internalize the intravascular fungal cell that had been arrested in the brain microvasculature and return to the blood stream in a “vacuum-cleaner” type of behavior	Zhang et al. (23)
*C. neoformans*	Zebrafish	The fungal cell was observed to proliferate within macrophages; capsule size determines early macrophage control of infection	Bojarczuk et al. (24)
*C. neoformans*	Zebrafish	The fungal cell can cross the zebrafish blood–brain barrier, which is dependent on the FNX1 virulence gene	Tenor et al. (25)
*C. albicans*	Mouse	Accumulation of both yeast and filamentous forms of the fungal cells were observed in the brain meninges and parenchyma	Navarathna et al. (26)
*P. brasiliensis*	Mouse	Enhanced leukocyte recruitment to the brain following the fungal infection is associated with CXCL9	Pedroso et al. (20)

## Intravital Microscopy

Intravital microscopy was first employed by Julius Cohnheim in the nineteenth century to visualize leukocyte trafficking in the tongue and mesentery of a frog (27). In the last decade, significant progress has been made in imaging of live animals due to breakthroughs in microscopy. Wide-field microscopy, multiphoton confocal, spinning disk confocal, and multiphoton resonant scanning confocal microscopy have been used to image fungal infection in the brain. Each imaging system has its advantages or disadvantages depending on whether speed of image acquisition, depth into the tissue, image resolution, photobleaching and phototoxicity, and price are considerations (28–32).

## IVM Procedure

There are two major surgical methods to make the brain vasculature visible under fluorescent microscopy, i.e., a thinned-skull cranial window and an open-skull cranial window (33). Both techniques have advantages and limitations. During imaging through the thinned-skull cranial window, the brain does not need to be superfused with artificial cerebrospinal fluids because the brain tissue is still covered with the skull. It is well suited for observations over long periods of time. However, the skull thickness affects the image quality and achieving optimal and uniform skull thickness requires a high level of surgical proficiency. By contrast, in an open-skull window, a portion of the skull and dura is removed, and the cortical surface is directly exposed to microscopy. Thus, a better quality of images is usually achieved compared with a thinned-skull window. However, it is essential to superfuse the brain with artificial cerebrospinal fluid during the period of observation, and great care must be taken to avoid surgical trauma and hemorrhage (33).

To facilitate intravital imaging, the organisms, brain microvasculature, and leukocytes can be labeled with fluorochromes. For example, we labeled *C. neoformans* with fluorescent isothiocyanate (FITC) or tetramethylrhodamine isothiocyanate (TRITC) to visualize the arrest and migration of the yeast cell into the brain (4, 5). Two colors allow comparison of two different virulence characteristics or wild-type and mutant strains. However, the yeast cell loses the fluorescent label if it proliferates. This disadvantage might be overcome by using fungi expressing green or red fluorescent proteins if sufficient fluorescent intensity can be achieved (26, 34, 35). To label the microvasculature, rat-anti-mouse PECAM-1 [CD31, a molecule expressed on endothelial cells (36)] can be injected intravenously (37, 38). Since the tight junctions of endothelial cells express high PECAM-1, this labeling can be used to study interactions of fungi or leukocytes with endothelial tight junctions (36). Alternatively, the vascular compartment can be illuminated by intravenous injection with fluorochrome-conjugated bovine serum albumin or dextran (39). In addition, transgenic mice that express fluorescent proteins in endothelial cells [for example, Tie-2 green fluorescent protein (GFP) mice (40)] can be used.

An expanding number of tools are becoming available to study the interactions of fungi with immune and inflammatory cells. To determine the trafficking of leukocytes in the brain, mice can be injected intravenously with rhodamine 6G, which is a cell-permeant dye that is sequestered by active mitochondria (41, 42). However, to identify the functions of subsets of leukocytes, mAb or transgenic mice can be used. For example, anti-CD45 can be injected intravenously, which labels all leukocytes. Neutrophils can be labeled *in vivo* by intravenous injection of anti-Ly6G (23). Alternatively, neutrophils can be visualized in mice expressing enhanced GFP under the control of the endogenous lysozyme promoter (LysM-eGFP) (39, 43). To image monocytes, mice can be intravenously injected with fluorochrome-labeled anti-CCR2 (labels proinflammatory monocytes) or anti-CX3CR1 antibody (labels patrolling monocytes) (44). Alternately, CX3CR1^gfp/+^ mice can be used to achieve this goal. In CX3CR1^gfp/+^ mice, one allele for the gene encoding CX3CR1, the receptor for chemokine CX3CL1, has been replaced with a gene encoding GFP, resulting in GFP expression of all circulating CD11b^+^F4/80^+^ cells. CX3CR1^gfp/+^ mice express GFP in monocytes, but not in neutrophils (45, 46). With time, many more mouse strains are becoming available that have fluorescent reporters linked to other genes that define different subsets of cells and allow us to study the role of those cells in the pathogenesis of infection.

## Brain Infection with *C. Neoformans*

*Cryptococcus neoformans* is an encapsulated budding yeast that causes a life-threatening illness in immunocompromised individuals, especially in AIDS patients. It is estimated that there are one million cases of cryptococcosis per year and 600,000 of these patients will die within 3 months of diagnosis (22). *Cryptococcus* is found in the environment and enters the body through the respiratory tract. Immunocompetent individuals are usually able to contain *C. neoformans* in the lung (47). In the case of an immunocompromised host, the yeast cells cannot be successfully contained and disseminate into the brain *via* the bloodstream, causing meningoencephalitis (16, 47).

Hematogenous dissemination of *C. neoformans* is one of the most critical steps in the development of meningoencephalitis. Prior to transmigration into the brain parenchyma, circulating *C. neoformans* must be arrested in the brain vasculature. We became interested in a number of questions related to the pathogenesis of cryptococcal meningoencephalitis. (1) Was *C. neoformans* arrested in the brain vasculature prior to transmigration and did the arrest occur in venules or capillaries? (2) How did *C. neoformans* behave during arrest? and (3) What was the mechanism(s) underlying the arrest of *C. neoformans*. As arrest of *C. neoformans* is a transient and dynamic process, we developed an *in vivo* model system based on IVM to study these questions (4, 5). We demonstrated that *C. neoformans* appeared in the mouse brain microvasculature within a few seconds after injection into the tail vein. When first seen, *C. neoformans* was moving with the same velocity as the blood, and no interaction of circulating *C. neoformans* with venular endothelial cells was observed. The number of yeast cells passing through postcapillary venules was greatest immediately after injection and gradually decreased over time. However, even after 18 h, rare yeast cells could still be seen moving in the brain venules. *C. neoformans* appeared to move at the same speed as the blood and came to a sudden stop in the capillaries of the brain without rolling and tethering to the endothelial surface. Interestingly, the yeast cells were arrested in capillaries that appeared to be of the same or smaller diameter than the organism, often at branch points. Differences in viability, polysaccharide capsule (the major virulence factor), and strain failed to affect the deposition of the yeast cells. In particular, there was no significant difference in the behavior and the arrest of polystyrene microspheres of similar size in the brain capillary bed when compared with *C. neoformans*. These results suggest that *C. neoformans* is mechanically trapped in the brain, which raises novel challenges for therapies to avoid arrest.

*Cryptococcus neoformans* transmigrates into the brain parenchyma across the BBB after arrest in the brain capillaries. Previous studies, using *in vitro* techniques, have shown that *C. neoformans* can cross the endothelium of the brain *via* direct transcytosis (6, 48, 49). It was further demonstrated that transcytosis is mediated by interactions between CD44 expressed on endothelium and cryptococcal hyaluronic acids (7, 8). A secreted fungal metalloprotease (9), an extracellular phospholipase B1 (10), and brain inositol (50) are critically involved in transcytosis of *C. neoformans*. In addition, it was also reported that *C. neoformans* invaded the brain *via* a “Trojan horse” mechanism with the help of phagocytes (11, 12). However, these studies have failed to determine the dynamics of BBB penetration by *C. neoformans* in the brain vasculature *in vivo*. Using IVM, we have recently characterized the transmigration of *C. neoformans in vivo* (5). Following arrest in the brain, *C. neoformans* was directly seen to cross the capillary wall of living animals in real time. In contrast to trapping, viability, but not replication, was required for *C. neoformans* to cross the BBB. Urease is critically involved in brain transmigration of the organism. Accordingly, a urease inhibitor could ameliorate infection of the mouse brain by reducing transmigration of *C. neoformans* into the brain, suggesting that a therapeutic strategy aimed at inhibiting this enzyme might be beneficial in cryptococcal meningitis and encephalitis.

Arrest of *C. neoformans* in the brain vasculature led to questions about recognition of the organism by circulating leukocytes. Recently, we addressed this question with the use of IVM (23). Among all subsets of leukocytes in the circulation, neutrophils are the most abundant phagocytes and are usually the first immune cells to be recruited to a site of infection to eliminate pathogens (51). Early work had suggested that human neutrophils kill *C. neoformans in vitro via* an intracellular (52, 53) or extracellular killing mechanism (54). In particular, the capability of human neutrophils to kill the organism was reported to be even greater than that of monocytes (52, 55). *In vitro*, mouse neutrophils appear to move toward *C. neoformans* and then rapidly internalize the yeast (56). Complement C5a–C5aR signaling was essential for phagocytosis of *C. neoformans* by neutrophils by guiding their migration to neutrophils and enhancing surface expression of CD11b (56). Furthermore, the p38 MAPK pathway, but not the Erk pathway, was critically involved in C5a–C5aR-mediated chemotaxis of neutrophils during their killing of *C. neoformans* (56). These *in vitro* observations encouraged us to address how neutrophils dynamically interact with *C. neoformans* which were arrested in the brain vasculature (23). With the use of IVM, we demonstrated that neutrophils crawled to the yeast cells that had been arrested in the brain microvasculature. Interestingly, crawling neutrophils recognized and interacted with the yeast, resulting in internalization of *C. neoformans*. During the interactions of neutrophils with the yeast, morphologic alterations of neutrophils, including deploying pseudopodia, were observed. Internalization of *C. neoformans* by neutrophils in the brain vasculature could be completed within a few minutes. Following ingestion of *C. neoformans*, neutrophils were seen to crawl again along the vessel wall and eventually to be released into the blood flow, resulting in a direct removal of the arrested *C. neoformans* from the brain vasculature. Depletion of neutrophils enhanced brain fungal burden (23), while enhancing the recruitment of neutrophils improved intravascular clearance of *C. neoformans* in the brain (57). Further studies demonstrated that *C. neoformans* infection led to enhanced expression of the adhesion molecule, Mac-1, on neutrophils, and ICAM-1 on brain endothelial cells. Complement C3 was critically involved in the recognition of *C. neoformans* by neutrophils and subsequent clearance of the organism from the brain (23). These results revealed that neutrophils are able to remove *C. neoformans* that had been arrested in the brain microvasculature in a “vacuum-cleaner” type of behavior. Given that neutrophils are usually considered to kill microorganisms at the infection site, the finding of the direct removal of *C. neoformans* by neutrophils from its arrested site may represent a novel mechanism of host defense in the brain (23). In this respect, neutrophils have been recently shown to “sweep up” bacteria arrested on the walls of an infected body cavity or blood vessel, but not fluid-borne bacteria in a zebrafish model (58).

Recently, a live-imaging model based on zebrafish larvae has been established to study the interactions of *C. neoformans* with innate immune cells and its migration to the brain (24, 25). The zebrafish *C. neoformans* platform provides a visually and genetically accessible vertebrate model system for infection of *C. neoformans*. It was shown that zebrafish macrophages rapidly phagocytosed the majority of *C. neoformans* cells following injection of the yeast *via* the caudal vein (25). Depletion of macrophages significantly enhanced the fungal burden in zebrafish, demonstrating that macrophages are essential to protect zebrafish from disease progression (24, 25). However, macrophages preferentially ingested *C. neoformans* with smaller polysaccharide capsules, and since the capsule size greatly increased over 24 h of infection, this markedly limited further phagocytosis (24). In addition, proliferation of *C. neoformans* within macrophages and non-lytic exocytosis of the yeast from macrophages were observed in zebrafish (24). Live imaging demonstrated that *C. neoformans* is able to penetrate the zebrafish brain. There was a positive correlation between the burden of organisms in cranial vessels versus invasion into the brain parenchyma (25). The *C. neoformans* fnx1Δ mutant, which is deficient in a multidrug resistance-like protein, was shown to have a deficiency in transmigration across the mouse BBB and reduced microvascular entrapment and transcytosis across immortalized human brain capillary endothelial cells *in vitro* (59). Interestingly, the fnx1Δ mutant also demonstrated defective invasion of brain parenchyma of zebrafish (25). Using IVM, these studies are just beginning to enhance our understanding of the spacial and temporal aspects and the role of different cell types in pathogenesis and host defense to *C. neoformans*.

## Brain Infection with *C. Albicans*

*Candida albicans* is a commensal organism and a common constituent of the normal mucosal flora. As the most common fungal pathogen of humans, overgrowth causes thrush. However, translocation of the yeast cells from the mucosal surface into the systemic circulation causes potentially life-threatening disease, particularly in post-surgical and critically ill patients, which is associated with approximately 35% death rate (60, 61). During this disease, the bloodborne organisms can spread to virtually all organs of the body. Although the kidney is the primary target of this organism during disseminated candidiasis, brain infection is found in approximately one-half of patients with systemic candidiasis at autopsy (62–64). In addition, *C. albicans* has also been reported to cause meningoencephalitis without systemic infection in healthy individuals (65).

To invade the brain parenchyma, circulating *C. albicans* cells must adhere and cross the BBB. Early work had shown that *C. albicans* is able to penetrate a monolayer of human brain endothelial cells cultured *in vitro via* a transcellular pathway (66). It was later demonstrated that *C. albicans* invasion of brain endothelial cells is mediated by the fungal invasins Als3 and Ssa1 (67). Als3 binds to the gp96 heat shock protein, a unique receptor that is expressed specifically on brain endothelium, promoting endothelial transcytosis by the fungus (67).

Recently, Navarathna et al. studied brain infection by *C. albicans* in a mouse model using IVM (26). They observed sporadic entry of *C. albicans* into the brain parenchyma as early as 30 min after intravenous inoculation. In this model, the authors did not observe leak of gadolinium diethylenetriaminepentaacetic acid (Gd-DTPA) into the brain 30 min after intravenous administration as examined by MRI, suggesting that brain invasion by *C. albicans* initially occurs without gross disruption of the BBB. However, IVM performed 3 days post-infection revealed significant accumulation of both yeast and filamentous forms of *C. albicans* in the meninges and parenchyma. At that time, leak of Gd-DTPA was observed, indicating damage of the BBB. The brain became heavily inflamed at sites of *C. albicans* invasion. Thus, it is conceivable that permeability of the BBB was caused by leukocyte infiltration. In addition, *Candida* filament elongation was observed in the brain. Interestingly, most of the yeast cells outside of the vasculature showed highly dynamic movement that could be explained by the movement of phagocytosed organisms within motile phagocytic cells. By contrast, hyphal cells showed only slow invasion based on hyphal extension.

## Brain Infection with *P. Brasiliensis*

*Paracoccidioides brasiliensis* is an etiologic agent of paracoccidioidomycosis, an important systemic mycosis in Latin America, with the greatest number of patients in Brazil, Venezuela, and Argentina (68). The infection is usually acquired by the respiratory system probably by inhalation of airborne conidia of *P. brasiliensis* (69, 70). Following infection, the conidia transform into yeast in the lungs. *P. brasiliensis* can cause disease in immunocompetent hosts, although immunosuppression increases the severity of infection. The yeast cells can be disseminated from the infected lung into other organs such as adrenal glands and brain (70, 71). In the last decade, brain infection has been reported more commonly, affecting approximately 12.5% of cases (70). However, it is unknown how the fungus arrests and migrates into the brain parenchyma across the BBB.

Recently, Pedroso et al. used IVM to examine trafficking of leukocytes in the brain in a murine model of neuroparacoccidioidomycosis (20). Following infection with *P. brasiliensis* by the intracranial route, mice showed clinical signs of progressive infection starting on day 7 post-inoculation. IVM of the brain pial microvasculature revealed a significant increase in leukocyte rolling 2 and 4 weeks post-infection and in adhesion 1, 2, and 4 weeks post-infection. The enhanced recruitment of leukocytes was associated with a significant increase in the brain concentration of chemokines, particularly CXCL9, suggesting a role for these molecules in the inflammatory and immune response against the fungi. The lesions were not restricted to the site of inoculation and disseminated to other sites of the brain including the cerebellum. Neutrophils and macrophages were increased in the brain as determined by the myeloperoxidase and *N*-acetyl-b-d-glucosaminidase activity in the brain tissues.

## Concluding Remarks

Fungal meningoencephalitis is a grave illness associated with high mortality, even with the best available antifungal treatment. Understanding the mechanisms involved in arrest and invasion of the brain by fungi and the interactions with immune cells is fundamental to our knowledge of the pathogenesis of the disease. With the use of IVM, brain infections by fungi, including *C. neoformans* (5, 23), *C. albicans* (26), and *P. brasiliensis* (20), have been recently investigated in real time. In particular, we have shown that *C. neoformans* is mechanically trapped in the brain vasculature (5). IVM may provide a powerful tool to determine whether *in vitro* findings implicating interactions between CD44 and hyaluronic acid (7, 8), or adherence of phagocytosed cells (Trojan Horse) also occur (11, 12) through the use of transgenic mice. Although neutrophils are able to recognize and remove the arrested *C. neoformans* from the brain vasculature (23), organisms were seen to cross the vessel wall with contribution of cryptococcal urease (5). IVM may provide a powerful tool to investigate the role of metalloprotease (9), and phospholipase (10) in brain invasion *via* transcytosis through the use of deletion mutants. The role of host immune cells in brain injury has been implicated in studies using *C. albicans*, and chemokines have been implicated by studies of *P. brasiliensis* (20, 26). In summary, recent advances in microscopy and the availability of antibody labeling *in vivo*, transgenic reporter mice and mice with targeted gene disruptions provide a powerful tool to examine fungal brain infections under more relevant physiologic conditions. It is expected that exciting findings regarding spacial and temporal aspects of pathogenic and immune mechanisms in fungal brain infections will be obtained with the application of IVM in the future, and that these insights may lead to new therapies.

## Author Contributions

All authors listed have made substantial, direct, and intellectual contribution to the work and approved it for publication.

## Conflict of Interest Statement

The authors declare that the research was conducted in the absence of any commercial or financial relationships that could be construed as a potential conflict of interest.

## References

[1] Kim KS. Mechanisms of microbial traversal of the blood-brain barrier. Nat Rev Microbiol (2008) 6(8):625–34. 10.1038/nrmicro1952 18604221 PMC5206914

[2] Owens T Bechmann I Engelhardt B. Perivascular spaces and the two steps to neuroinflammation. J Neuropathol Exp Neurol (2008) 67(12):1113–21. 10.1097/NEN.0b013e31818f9ca8 19018243

[3] Engelhardt B Sorokin L. The blood-brain and the blood-cerebrospinal fluid barriers: function and dysfunction. Semin Immunopathol (2009) 31(4):497–511. 10.1007/s00281-009-0177-0 19779720

[4] Shi M Colarusso P Mody CH Real-time in vivo imaging of fungal migration to the central nervous system. Cell Microbiol (2012) 14(12):1819–27. 10.1111/cmi.12027 22966777

[5] Shi M Li SS Zheng C Jones GJ Kim KS Zhou H Real-time imaging of trapping and urease-dependent transmigration of *Cryptococcus neoformans* in mouse brain. J Clin Invest (2010) 120(5):1683–93. 10.1172/JCI41963 20424328 PMC2860939

[6] Chang YC Stins MF McCaffery MJ Miller GF Pare DR Dam T Cryptococcal yeast cells invade the central nervous system via transcellular penetration of the blood-brain barrier. Infect Immun (2004) 72(9):4985–95. 10.1128/IAI.72.9.4985-4995.2004 15321990 PMC517459

[7] Jong A Wu CH Gonzales-Gomez I Kwon-Chung KJ Chang YC Tseng HK Hyaluronic acid receptor CD44 deficiency is associated with decreased *Cryptococcus neoformans* brain infection. J Biol Chem (2012) 287(19):15298–306. 10.1074/jbc.M112.353375 22418440 PMC3346080

[8] Jong A Wu CH Shackleford GM Kwon-Chung KJ Chang YC Chen HM Involvement of human CD44 during *Cryptococcus neoformans* infection of brain microvascular endothelial cells. Cell Microbiol (2008) 10(6):1313–26. 10.1111/j.1462-5822.2008.01128.x 18248627

[9] Vu K Tham R Uhrig JP Thompson GR III Na Pombejra S Jamklang M Invasion of the central nervous system by *Cryptococcus neoformans* requires a secreted fungal metalloprotease. MBio (2014) 5(3):e1101–14. 10.1128/mBio.01101-14 24895304 PMC4049100

[10] Maruvada R Zhu L Pearce D Zheng Y Perfect J Kwon-Chung KJ *Cryptococcus neoformans* phospholipase B1 activates host cell Rac1 for traversal across the blood-brain barrier. Cell Microbiol (2012) 14(10):1544–53. 10.1111/j.1462-5822.2012.01819.x 22646320 PMC3443264

[11] Charlier C Nielsen K Daou S Brigitte M Chretien F Dromer F. Evidence of a role for monocytes in dissemination and brain invasion by *Cryptococcus neoformans*. Infect Immun (2009) 77(1):120–7. 10.1128/IAI.01065-08 18936186 PMC2612285

[12] Sorrell TC Juillard PG Djordjevic JT Kaufman-Francis K Dietmann A Milonig A Cryptococcal transmigration across a model brain blood-barrier: evidence of the Trojan horse mechanism and differences between *Cryptococcus neoformans var. grubii* strain H99 and *Cryptococcus gattii* strain R265. Microbes Infect (2016) 18(1):57–67. 10.1016/j.micinf.2015.08.017 26369713

[13] Bryan AM Del Poeta M Luberto C. Sphingolipids as regulators of the phagocytic response to fungal infections. Mediators Inflamm (2015) 2015:640540. 10.1155/2015/640540 26688618 PMC4673356

[14] Bariola JR Perry P Pappas PG Proia L Shealey W Wright PW Blastomycosis of the central nervous system: a multicenter review of diagnosis and treatment in the modern era. Clin Infect Dis (2010) 50(6):797–804. 10.1086/650579 20166817

[15] Chakrabarti A. Epidemiology of central nervous system mycoses. Neurol India (2007) 55(3):191–7. 10.4103/0028-3886.35679 17921647

[16] Gottfredsson M Perfect JR. Fungal meningitis. Semin Neurol (2000) 20(3):307–22. 10.1055/s-2000-9394 11051295

[17] Kleinschmidt-DeMasters BK. Central nervous system aspergillosis: a 20-year retrospective series. Hum Pathol (2002) 33(1):116–24. 10.1053/hupa.2002.30186 11823982

[18] Liu TB Perlin DS Xue C. Molecular mechanisms of cryptococcal meningitis. Virulence (2012) 3(2):173–81. 10.4161/viru.18685 22460646 PMC3396696

[19] Murthy JM. Fungal infections of the central nervous system: the clinical syndromes. Neurol India (2007) 55(3):221–5. 10.4103/0028-3886.35682 17921650

[20] Pedroso VS Vilela MC Santos PC Cisalpino PS Rachid MA Teixeira AL. Traffic of leukocytes and cytokine up-regulation in the central nervous system in a murine model of neuroparacoccidioidomycosis. Mycopathologia (2013) 176(3–4):191–9. 10.1007/s11046-013-9679-3 23877333

[21] Scully EP Baden LR Katz JT. Fungal brain infections. Curr Opin Neurol (2008) 21(3):347–52. 10.1097/WCO.0b013e3282fee95b 18451721

[22] Park BJ Wannemuehler KA Marston BJ Govender N Pappas PG Chiller TM. Estimation of the current global burden of cryptococcal meningitis among persons living with HIV/AIDS. AIDS (2009) 23(4):525–30. 10.1097/QAD.0b013e328322ffac 19182676

[23] Zhang M Sun D Liu G Wu H Zhou H Shi M. Real-time in vivo imaging reveals the ability of neutrophils to remove *Cryptococcus neoformans* directly from the brain vasculature. J Leukoc Biol (2016) 99(3):467–73. 10.1189/jlb.4AB0715-281R 26428677 PMC6608047

[24] Bojarczuk A Miller KA Hotham R Lewis A Ogryzko NV Kamuyango AA *Cryptococcus neoformans* intracellular proliferation and capsule size determines early macrophage control of infection. Sci Rep (2016) 6:21489. 10.1038/srep21489 26887656 PMC4757829

[25] Tenor JL Oehlers SH Yang JL Tobin DM Perfect JR. Live imaging of host-parasite interactions in a zebrafish infection model reveals cryptococcal determinants of virulence and central nervous system invasion. MBio (2015) 6(5):e1425–1415. 10.1128/mBio.01425-15 26419880 PMC4611042

[26] Navarathna DH Munasinghe J Lizak MJ Nayak D McGavern DB Roberts DD. MRI confirms loss of blood-brain barrier integrity in a mouse model of disseminated candidiasis. NMR Biomed (2013) 26(9):1125–34. 10.1002/nbm.2926 23606437 PMC3744627

[27] Dutrochet H Recherches Anatomiques et Physiologiques sur la Structure Intime des Animaux et des Vegetaux, et sur Leur Motilite. Paris: Bailliere et fils (1824). PMC5754632 30329773

[28] Helmchen F Denk W. Deep tissue two-photon microscopy. Nat Methods (2005) 2(12):932–40. 10.1038/nmeth818 16299478

[29] Graf R Rietdorf J Zimmermann T. Live cell spinning disk microscopy. Adv Biochem Eng Biotechnol (2005) 95:57–75. 10.1007/b102210 16080265

[30] Murray JM. Methods for imaging thick specimens: confocal microscopy, deconvolution, and structured illumination. Cold Spring Harb Protoc (2011) 2011(12):1399–437. 10.1101/pdb.top066936 22135661

[31] Conchello JA Lichtman JW. Optical sectioning microscopy. Nat Methods (2005) 2(12):920–31. 10.1038/nmeth815 16299477

[32] Ustione A Piston DW A simple introduction to multiphoton microscopy. J Microsc (2011) 243(3):221–6. 10.1111/j.1365-2818.2011.03532.x 21777244

[33] Yang G Pan F Parkhurst CN Grutzendler J Gan WB. Thinned-skull cranial window technique for long-term imaging of the cortex in live mice. Nat Protoc (2010) 5(2):201–8. 10.1038/nprot.2009.222 20134419 PMC4690457

[34] Lionakis MS Swamydas M Fischer BG Plantinga TS Johnson MD Jaeger M CX3CR1-dependent renal macrophage survival promotes *Candida* control and host survival. J Clin Invest (2013) 123(12):5035–51. 10.1172/JCI71307 24177428 PMC3859390

[35] Voelz K Johnston SA Rutherford JC May RC. Automated analysis of cryptococcal macrophage parasitism using GFP-tagged cryptococci. PLoS One (2010) 5(12):e15968. 10.1371/journal.pone.0015968 21209844 PMC3013146

[36] Albelda SM Muller WA Buck CA Newman PJ. Molecular and cellular properties of PECAM-1 (endoCAM/CD31): a novel vascular cell-cell adhesion molecule. J Cell Biol (1991) 114(5):1059–68. 10.1083/jcb.114.5.1059 1874786 PMC2289123

[37] Moriarty TJ Norman MU Colarusso P Bankhead T Kubes P Chaconas G. Real-time high resolution 3D imaging of the Lyme disease spirochete adhering to and escaping from the vasculature of a living host. PLoS Pathog (2008) 4(6):e1000090. 10.1371/journal.ppat.1000090 18566656 PMC2408724

[38] Phillipson M Heit B Colarusso P Liu L Ballantyne CM Kubes P. Intraluminal crawling of neutrophils to emigration sites: a molecularly distinct process from adhesion in the recruitment cascade. J Exp Med (2006) 203(12):2569–75. 10.1084/jem.20060925 17116736 PMC2118150

[39] McDonald B Pittman K Menezes GB Hirota SA Slaba I Waterhouse CC Intravascular danger signals guide neutrophils to sites of sterile inflammation. Science (2010) 330(6002):362–6. 10.1126/science.1195491 20947763

[40] Motoike T Loughna S Perens E Roman BL Liao W Chau TC Universal GFP reporter for the study of vascular development. Genesis (2000) 28(2):75–81. 10.1002/1526-968X(200010)28:2<75::AID-GENE50>3.0.CO;2-S 11064424

[41] Zhou H Andonegui G Wong CH Kubes P. Role of endothelial TLR4 for neutrophil recruitment into central nervous system microvessels in systemic inflammation. J Immunol (2009) 183(8):5244–50. 10.4049/jimmunol.0901309 19786543

[42] Zhou H Lapointe BM Clark SR Zbytnuik L Kubes P. A requirement for microglial TLR4 in leukocyte recruitment into brain in response to lipopolysaccharide. J Immunol (2006) 177(11):8103–10. 10.4049/jimmunol.177.11.8103 17114485

[43] Faust N Varas F Kelly LM Heck S Graf T. Insertion of enhanced green fluorescent protein into the lysozyme gene creates mice with green fluorescent granulocytes and macrophages. Blood (2000) 96(2):719–26. 10887140

[44] Auffray C Fogg D Garfa M Elain G Join-Lambert O Kayal S Monitoring of blood vessels and tissues by a population of monocytes with patrolling behavior. Science (2007) 317(5838):666–70. 10.1126/science.1142883 17673663

[45] Geissmann F Jung S Littman DR. Blood monocytes consist of two principal subsets with distinct migratory properties. Immunity (2003) 19(1):71–82. 10.1016/S1074-7613(03)00174-2 12871640

[46] Jung S Aliberti J Graemmel P Sunshine MJ Kreutzberg GW Sher A Analysis of fractalkine receptor CX(3)CR1 function by targeted deletion and green fluorescent protein reporter gene insertion. Mol Cell Biol (2000) 20(11):4106–14. 10.1128/MCB.20.11.4106-4114.2000 10805752 PMC85780

[47] Kwon-Chung KJ Sorrell TC Dromer F Fung E Levitz SM. Cryptococcosis: clinical and biological aspects. Med Mycol (2000) 38(Suppl 1):205–13. 10.1080/mmy.38.s1.205.213 11204147

[48] Sabiiti W May RC. Capsule independent uptake of the fungal pathogen *Cryptococcus neoformans* into brain microvascular endothelial cells. PLoS One (2012) 7(4):e35455. 10.1371/journal.pone.0035455 22530025 PMC3328471

[49] Vu K Weksler B Romero I Couraud PO Gelli A. Immortalized human brain endothelial cell line HCMEC/D3 as a model of the blood-brain barrier facilitates in vitro studies of central nervous system infection by *Cryptococcus neoformans*. Eukaryot Cell (2009) 8(11):1803–7. 10.1128/EC.00240-09 19767445 PMC2772405

[50] Liu TB Kim JC Wang Y Toffaletti DL Eugenin E Perfect JR Brain inositol is a novel stimulator for promoting *Cryptococcus* penetration of the blood-brain barrier. PLoS Pathog (2013) 9(4):e1003247. 10.1371/journal.ppat.1003247 23592982 PMC3617100

[51] Kolaczkowska E Kubes P. Neutrophil recruitment and function in health and inflammation. Nat Rev Immunol (2013) 13(3):159–75. 10.1038/nri3399 23435331

[52] Diamond RD Root RK Bennett JE Factors influencing killing of *Cryptococcus neoformans* by human leukocytes in vitro. J Infect Dis (1972) 125(4):367–76. 10.1093/infdis/125.4.367 4553080

[53] Kozel TR Highison B Stratton CJ. Localization on encapsulated *Cryptococcus neoformans* of serum components opsonic for phagocytosis by macrophages and neutrophils. Infect Immun (1984) 43(2):574–9. 6363293 10.1128/iai.43.2.574-579.1984 PMC264336

[54] Qureshi A Subathra M Grey A Schey K Del Poeta M Luberto C. Role of sphingomyelin synthase in controlling the antimicrobial activity of neutrophils against *Cryptococcus neoformans*. PLoS One (2010) 5(12):e15587. 10.1371/journal.pone.0015587 21203393 PMC3011003

[55] Miller MF Mitchell TG. Killing of *Cryptococcus neoformans* strains by human neutrophils and monocytes. Infect Immun (1991) 59(1):24–8. 1987038 10.1128/iai.59.1.24-28.1991 PMC257700

[56] Sun D Zhang M Liu G Wu H Zhu X Zhou H Real-time imaging of interactions of neutrophils with *Cryptococcus neoformans* demonstrates a crucial role of complement C5a-C5aR signaling. Infect Immun (2015) 84(1):216–29. 10.1128/IAI.01197-15 26502909 PMC4693990

[57] Sun D Zhang M Liu L Wu H Li C Zhou H Intravascular clearance of disseminating *Cryptococcus neoformans* in the brain can be improved by enhancing neutrophil recruitment in mice. Eur J Immunol (2016) 46(7):1704–14. 10.1002/eji.201546239 27109176 PMC5165700

[58] Colucci-Guyon E Tinevez JY Renshaw SA Herbomel P. Strategies of professional phagocytes in vivo: unlike macrophages, neutrophils engulf only surface-associated microbes. J Cell Sci (2011) 124(Pt 18):3053–9. 10.1242/jcs.082792 21868367

[59] Tseng HK Liu CP Price MS Jong AY Chang JC Toffaletti DL Identification of genes from the fungal pathogen *Cryptococcus neoformans* related to transmigration into the central nervous system. PLoS One (2012) 7(9):e45083. 10.1371/journal.pone.0045083 23028773 PMC3447876

[60] Horn DL Neofytos D Anaissie EJ Fishman JA Steinbach WJ Olyaei AJ Epidemiology and outcomes of candidemia in 2019 patients: data from the prospective antifungal therapy alliance registry. Clin Infect Dis (2009) 48(12):1695–703. 10.1086/599039 19441981

[61] Wisplinghoff H Bischoff T Tallent SM Seifert H Wenzel RP Edmond MB. Nosocomial bloodstream infections in US hospitals: analysis of 24,179 cases from a prospective nationwide surveillance study. Clin Infect Dis (2004) 39(3):309–17. 10.1086/421946 15306996

[62] Parker JC Jr McCloskey JJ Lee RS Human cerebral candidosis – a postmortem evaluation of 19 patients. Hum Pathol (1981) 12(1):23–8. 10.1016/S0046-8177(81)80238-9 7203451

[63] Pendlebury WW Perl DP Munoz DG. Multiple microabscesses in the central nervous system: a clinicopathologic study. J Neuropathol Exp Neurol (1989) 48(3):290–300. 10.1097/00005072-198905000-00006 2649643

[64] Sanchez-Portocarrero J Perez-Cecilia E Corral O Romero-Vivas J Picazo JJ. The central nervous system and infection by *Candida* species. Diagn Microbiol Infect Dis (2000) 37(3):169–79. 10.1016/S0732-8893(00)00140-1 10904190

[65] Borha A Parienti JJ Emery E Coskun O Khouri S Derlon JM. [*Candida albicans* cerebral granuloma in an immunocompetent patient. A case report]. Neurochirurgie (2009) 55(1):57–62. 10.1016/j.neuchi.2008.06.001 18692208

[66] Jong AY Stins MF Huang SH Chen SH Kim KS. Traversal of *Candida albicans* across human blood-brain barrier in vitro. Infect Immun (2001) 69(7):4536–44. 10.1128/IAI.69.7.4536-4544.2001 11401997 PMC98530

[67] Liu Y Mittal R Solis NV Prasadarao NV Filler SG. Mechanisms of *Candida albicans* trafficking to the brain. PLoS Pathog (2011) 7(10):e1002305. 10.1371/journal.ppat.1002305 21998592 PMC3188548

[68] de Oliveira HC Assato PA Marcos CM Scorzoni L de Paula ESAC Da Silva Jde F Paracoccidioides-host interaction: an overview on recent advances in the paracoccidioidomycosis. Front Microbiol (2015) 6:1319. 10.3389/fmicb.2015.01319 26635779 PMC4658449

[69] McEwen JG Bedoya V Patino MM Salazar ME Restrepo A. Experimental murine paracoccidiodomycosis induced by the inhalation of conidia. J Med Vet Mycol (1987) 25(3):165–75. 10.1080/02681218780000231 3612432

[70] Pedroso VS Vilela Mde C Pedroso ER Teixeira AL. [Paracoccidioidomycosis compromising the central nervous system: a systematic review of the literature]. Rev Soc Bras Med Trop (2009) 42(6):691–7. 10.1590/S0037-86822009000600016 20209357

[71] Paniago AM de Oliveira PA Aguiar ES Aguiar JI da Cunha RV Leme LM Neuroparacoccidioidomycosis: analysis of 13 cases observed in an endemic area in Brazil. Trans R Soc Trop Med Hyg (2007) 101(4):414–20. 10.1016/j.trstmh.2006.07.006 17011605

